# Enhancing the Oncolytic Activity of CD133-Targeted Measles Virus: Receptor Extension or Chimerism with Vesicular Stomatitis Virus Are Most Effective

**DOI:** 10.3389/fonc.2017.00127

**Published:** 2017-06-26

**Authors:** Dina Kleinlützum, Julia D. S. Hanauer, Alexander Muik, Kay-Martin Hanschmann, Sarah-Katharina Kays, Camilo Ayala-Breton, Kah-Whye Peng, Michael D. Mühlebach, Tobias Abel, Christian J. Buchholz

**Affiliations:** ^1^Molecular Biotechnology and Gene Therapy, Paul-Ehrlich-Institut, Langen, Germany; ^2^German Cancer Consortium (DKTK), Partner Site Heidelberg, Heidelberg, Germany; ^3^German Cancer Research Center (DKFZ), Heidelberg, Germany; ^4^Biostatistics, Paul-Ehrlich-Institut, Langen, Germany; ^5^Department of Molecular Medicine, Mayo Clinic, Rochester, MN, United States; ^6^Product Testing of Immunological Medicinal Products for Veterinary Use, Paul-Ehrlich-Institut, Langen, Germany

**Keywords:** glioblastoma, hepatocellular carcinoma, prominin-1, virotherapy, tumorsphere

## Abstract

Therapy resistance and tumor recurrence are often linked to a small refractory and highly tumorigenic subpopulation of neoplastic cells, known as cancer stem cells (CSCs). A putative marker of CSCs is CD133 (prominin-1). We have previously described a CD133-targeted oncolytic measles virus (MV-CD133) as a promising approach to specifically eliminate CD133-positive tumor cells. Selectivity was introduced at the level of cell entry by an engineered MV hemagglutinin (H). The H protein was blinded for its native receptors and displayed a CD133-specific single-chain antibody fragment (scFv) as targeting domain. Interestingly, MV-CD133 was more active in killing CD133-positive tumors than the unmodified MV-NSe despite being highly selective for its target cells. To further enhance the antitumoral activity of MV-CD133, we here pursued arming technologies, receptor extension, and chimeras between MV-CD133 and vesicular stomatitis virus (VSV). All newly generated viruses including VSV-CD133 were highly selective in eliminating CD133-positive cells. MV-CD46/CD133 killed in addition CD133-negative cells being positive for the MV receptors. In an orthotopic glioma model, MV-CD46/CD133 and MV^SCD^-CD133, which encodes the super cytosine deaminase, were most effective. Notably, VSV-CD133 caused fatal neurotoxicity in this tumor model. Use of CD133 as receptor could be excluded as being causative. In a subcutaneous tumor model of hepatocellular cancer, VSV-CD133 revealed the most potent oncolytic activity and also significantly prolonged survival of the mice when injected intravenously. Compared to MV-CD133, VSV-CD133 infected a more than 10^4^-fold larger area of the tumor within the same time period. Our data not only suggest new concepts and approaches toward enhancing the oncolytic activity of CD133-targeted oncolytic viruses but also raise awareness about careful toxicity testing of novel virus types.

## Introduction

Despite considerable progress in cancer therapy, relapse and dissemination of tumor cells remain a frequent therapeutic outcome, which is more and more ascribed to an insufficient targeting and killing of a small population of tumor cells with stem-cell like properties (1). Such cancer stem cells (CSCs) were initially detected in tumors of hematopoietic origin demonstrating that only a small fraction of cells in the tumor mass is capable of forming metastasis and new tumors (2). Among many putative markers for CSCs, CD133 was among the first to be discovered in carcinomas and since then became one of the most frequently studied and targeted marker (3, 4). Besides on tumor cells, CD133 is expressed on neuronal and endothelial progenitors as well as on hematopoietic stem cells (HSCs). While there is evidence for a role of CD133 in cell differentiation and epidermal–mesenchymal transition, its precise physiological function remains unknown (4).

The relevance of CD133 as universal marker for CSCs has frequently been challenged so that there is ample evidence for the presence of stemness properties also in CD133-negative tumor cells (5). Nevertheless, evidence for a strong correlation between high levels of CD133 expression and poor prognosis for patients suffering from various cancer types has increased, as well. In glioma, a systematic meta-analysis covering 1,500 patients revealed reduced overall survival for grade IV patients with high CD133 expression (6). Since further recent articles came to the same conclusion for glioma (7–9) as for hepatocellular cancer (HCC) (10, 11), targeting of CD133 remains an attractive therapeutic concept for these cancer entities and possibly also for others.

Oncolytic viruses have become a novel treatment option in cancer therapy with a first product based on herpes virus having recently obtained marketing approval (12). The antitumor concept relies on the selective infection and lysis of tumor cells resulting in the release of tumor antigens against which an effective immune response can be triggered (13). Oncolytic measles viruses (MVs) derived from attenuated strains are currently studied in various clinical trials assessing their antitumoral activity for different cancer entities (14, 15). Recently, clinical benefit was achieved for patients suffering from multiple myeloma upon systemic injection of a high MV dose (16). Supposedly, selectivity for tumor cells is on one hand due to overexpression of CD46, one of the receptors used by attenuated MV strains. On the other hand, attenuated MV strains are sensitive to much lower levels of interferon than wild-type MV strains, a phenomenon thought to be a consequence of mutations in the P/V/C gene that accumulated during attenuation (17, 18). Interestingly, the oncolytic activity of MV can be enhanced by exchanging the P gene from attenuated strains against that of wild-type MV without compromising safety (19).

Restriction of virus replication to tumor cells can moreover be achieved through rational engineering at the level of receptor recognition and cell entry. In particular, the glycoproteins of herpes simplex virus and MV have been shown to be amenable for engineering receptor usage (20, 21). In the case of MV, recognition of the natural receptors CD46, SLAM, and nectin-4 (22) can be destroyed by four point mutations and new receptor usage gained by fusing a single-chain antibody fragment (scFv) or other targeting domains to the hemagglutinin on the genetic level (23, 24). Such MVs only infect cells that express the cognate antigen of the displayed targeting domain on their surface (25). Interestingly, the MV glycoproteins can replace the glycoprotein G in the vesicular stomatitis virus (VSV) genome, resulting in chimeric VSV-MVs that replicate faster and to higher titers than the corresponding MVs (26).

By displaying CD133-recognizing scFvs, we have previously shown that oncolytic MV can be engineered to recognize CD133 as receptor for cell entry and can selectively destroy CD133-positive tumor cells (27). Remarkably, these viruses exhibited a stronger oncolytic activity *in vivo* than untargeted MV using the ubiquitously expressed CD46 receptor for cell entry. In a clinical setting, CSCs are rare in tumor tissue making it challenging for CD133-targeted viruses to hit and infect these cells. Here, we therefore aimed at further improving the oncolytic activity of CD133-targeted oncolytic viruses by assessing various strategies. We show that in glioma, MVs using CD133 and CD46 as receptors are particularly promising, while for HCC or other carcinomas not involving the central nervous system, VSV targeted to CD133 appears to be the best choice.

## Materials and Methods

### Generation of the Viruses

Cloning of MV-CD133, previously termed MV-141.7, was described before (27). To generate MV^Pwt^-CD133 the reading frame for H in the plasmid encoding MV-eGFP-Pwt (19) was exchanged against that of the engineered H protein encoded in the genome of MV-CD133 *via* PacI/SpeI restriction sites. MV^SCD^-CD133 was generated by exchanging the GFP coding sequence in the genome of MV-CD133 against that of SCD using the MluII/AatII restriction sites. To reconstitute the N and P genes after SCD insertion, these were inserted *via* AatII restriction in a second step. The genome of MV-CD46/CD133 was cloned by first generating the expression plasmid pCG-H-scFvCD133-141.7-6His encoding the H protein C-terminally fused to the CD133-specific scFv 141.7 (27), but carrying no point mutations in the MV-receptor recognition sites. The H gene cassette in MV-CD133 was then exchanged against that of pCG-H-scFvCD133-141.7-6His *via* PacI/SpeI restriction sites. Interested researchers may request Miltenyi Biotec GmbH (Germany) to grant access to the plasmids under a Material Transfer Agreement.

Rescue of MV-CD133, MV^Pwt^-CD133, MV^SCD^-CD133, and MV-CD46/CD133 was performed using the T7 rescue system with 293-3-46 producer cells (28) overlaid onto Vero-αHis cells (25). Starting from a single virus syncytium, virus was propagated on Vero-αHis cells and stocks were generated from cell lysates.

For cloning of the genome plasmid of VSV-CD133, the sequence encoding the CD133-specific scFv was inserted into pMC11-VSV-FHaa-mUPA-eGFP (encodes the non-attenuated Indiana serotype) *via* SfiI/NotI restriction sites (29). To rescue VSV-CD133, in addition to the helper plasmids encoding VSV-N, -P and -L, a plasmid encoding VSV-G was co-transfected into BHK-21 cells. The T7 RNA polymerase was provided by infection of the transfected BHK-21 cells with a modified vaccinia virus Ankara coding for the polymerase (MVA-T7-Pol) (30). Cell lysate was harvested, MVA was removed by filtration (0.2 µm pores), and single syncytia were isolated after overlay on Vero-αHis cells as described. VSV-MV was rescued from pMC11-VSVFH-eGFP as described previously (26). VSV-CD133 and VSV-MV were propagated on Vero-αHis cells. The 50% tissue culture infective dose (TCID_50_/ml) was determined on Vero-αHis cells.

All viruses were handled under biosafety level 2 conditions as authorized by the Regierungspräsidium Giessen, Germany.

### Cells

BHK-21 (ATCC CCL-10), Chinese hamster ovary (CHO)-K1 (ATCC CCL-61) cells, HuH7 cells (Japanese Collection of Research Bioresources Cell Bank, Japan), 293-3-46 cells (28), Vero-αHis cells (25), CHO-CD46 cells (31), and CHO-hSLAM cells (32) were all cultivated in DMEM (Sigma-Aldrich, Germany) supplemented with 10% FCS (Biochrom, Germany) and 2 mM l-glutamine (Sigma-Aldrich, Germany). CHO-CD133 cells were generated by stable integration of the human CD133 coding sequence into CHO-K1 cells (ATCC CCL-61). The cells were cultivated in DMEM supplemented with 10% FCS and 10 µg/ml puromycin.

Primary glioblastoma cells NCH644 and human HSCs were cultivated as described previously (27).

### Immunoblotting

Virus stocks (5.0 × 10^5^ TCID_50_: MV-NSe, MV-CD133, MV^Pwt^-CD133, MV^SCD^-CD133, MV-CD46/CD133; 2.5 × 10^5^ TCID_50_: VSV-MV and VSV-CD133) were mixed with urea sample buffer (5% SDS, 8 mM urea, 200 mM Tris–HCl, 0.1 mM EDTA, 0.03% bromphenol blue, 2.5% di-thiothreitol, pH 8.0) in equivalent amounts and incubated 10 min at 95°C before separating them *via* SDS-PAGE. Proteins were blotted onto a nitrocellulose membrane (GE Healthcare, Germany) and blocked with TBS-T containing 5% milk powder. Subsequently, membranes were incubated with rabbit sera recognizing MV-F (Abcam, Great Britain), the cytoplasmic tail of MV-H (27), MV-N (Novus Biologicals, USA), or rabbit-α-VSV serum (α-VSV) as described in Ref. (33). After three wash steps, membranes were incubated with polyclonal horseradish peroxidase-conjugated goat-α-rabbit secondary antibody (DakoCytomation, Germany). Protein signals were detected using the Pierce ECL Plus Western Blotting Substrate (Thermo Fisher Scientific, USA).

### Colony-Forming Assays

Primary human CD34-positive cells derived from G-CSF mobilized peripheral blood of anonymous donors were obtained from the blood donation center in Frankfurt in accordance with the ethical standards of the responsible committee on human experimentation. An ethics approval was not needed for this type of research. The cells were purified and cultivated as described (34). After overnight stimulation with medium supplemented with StemSpan CC100 cytokine cocktail (Stemcell Technologies, Germany) and 2 mg/mL TPO (Peprotech, Rocky Hill, NJ), 5 × 10^4^ cells were infected with virus at an MOI of 1. Lysate of uninfected Vero-αHis cells was added as control and to equilibrate all samples to identical amounts of cell lysate. 24 h postinfection, 0.1 mM 5-fluorocytosine was added to the cells. 48 h postinfection, cells were washed twice with medium without cytokines. Next, 1% of the cells were transferred into 3 ml MethoCult GF H4434 medium (Stemcell Technologies, Germany) and plated in triplicates. After 10 days in an incubator at 37°C and 95% humidity, clonal clusters (colonies) of maturing cells of the myeloid and erythroid lineage were enumerated and morphologically classified by light microscopy.

### Monitoring Cell Viability

For analyzing the viability of virus-infected HuH7 cells, 1.0 × 10^4^ cells were seeded per 96-well and infected at different MOIs (0.0001–10) to determine dose dependency. Cell viability of infected cells was determined using the premixed WST-1 Cell Proliferation Reagent (Clontech, USA) according the manufacturer protocol. To determine the EC_50_, the MOI required to kill half of the cells was determined for each virus.

To analyze cell viability in glioma sphere cultures, 7.0 × 10^3^ NCH644 cells were seeded per well in a 96-well plate and infected with different MOIs (0.001–10) to determine dose dependency. When using MV^SCD^-CD133, different concentrations of 5-FC were added to the culture medium to determine the optimal dose response. Cell viability of infected cells was determined using the RealTime-Glo MT Cell Viability assay (Promega, Germany) according to the manufacturer’s protocol.

To determine IFNα in the supernatant of infected cells, cell culture supernatants were collected at time points 0, 12, 24, 48, and 72 h postinfection. Cell free supernatants were obtained by centrifugation at 250 *g* and stored at −80°C until analysis by Human IFN-α pan ELISA (Mabtech, Sweden) according to the manufacturer’s instructions.

### Orthotopic Glioma Model

1 × 10^5^ NCH644 tumor sphere cells either uninfected or infected at an MOI of 0.5 were dissociated and stereotactically implanted in 5 µl of PBS into the corpus striatum of the right hemisphere (1.7 mm lateral, 0.5 mm rostral to bregma at 3 mm depth) of 6- to 8-week-old NOD/SCID mice. After pausing for 5 min to allow the diffusion of the carrier fluid into the brain parenchyma, the injection needle was slowly extracted. Intracranial injection of virus was performed stereotactically in 5 µl PBS into the same coordinates. Survival and general condition of mice were monitored daily. The experimental end point was reached at the onset of neurological symptoms and/or weight loss of more than 20%.

For *ex vivo* culturing of glioma spheres, the whole tumor was excised and enzymatically dissociated using the Miltenyi Brain tumor dissociation Kit according to the manufacturers’ instructions. 2 × 10^5^ single cells were cultured in T25 flask (Sarstedt, Germany) in DMEM/F-12 medium containing 20% BIT serum-free supplement, basic fibroblast growth factor, and epidermal growth factor at 20 ng/mL (all Provitro, Germany). Cells were cultivated until spheres had formed, and a cell density of about 1 × 10^6^ cells per T25 flask was reached.

### HuH7 Tumor Model

To analyze the antitumoral effect of oncolytic viruses in the s.c. HuH7 xenograft model, 5 × 10^6^ HuH7 cells were implanted into the flank of NOD/SCID mice (Charles River, Germany). The tumor development was monitored twice a week using a digital caliper. Once tumors had reached an average size of 100 mm^3^ mice were assigned into the different treatment groups. A total dose of 4 × 10^6^ TCID_50_ of each virus in 50 µl Vero-αHis lysate was injected intratumorally split in four injections every other day. Control mice received virus-free Vero-αHis lysate. For intravenous administration 3 × 50 µl virus were injected *via* the tail vein every second day. Treatment and monitoring were performed in a blinded manner over the whole course of the study until sacrifice. The area under the curve (AUC) was determined for each animal and was normalized against the value obtained from the last survivor of the mock group (day 40 posttransplantation). To account for missing scores of the tumor size of sacrificed animals before the end point for the analysis, the values were carried forward until termination of the study (last observation carried forward). NOD/SCID mice were sacrificed once the tumor had reached a size of 1,000 mm^3^ or mice had lost more than 20% of their body weight.

### Quantification of Infected Areas in Tumor Sections

HuH7-derived tumors were explanted, cut into two halves and fixed in 4% formaldehyde in PBS for 24 h. Next, the tissue was dehydrated in 40% sucrose in PBS and then embedded in optical cutting temperature medium (Sakura Finetek, Germany) for snap freezing. Specimens were stored at −80°C. Slices of 8 µm thickness were obtained with a cryostat (Leica CM1900) and dried at room temperature overnight. Slices were permeabilized with 0.2% Triton X100/PBS for 10 min and blocked with 5% donkey serum for 30 min at room temperature. For staining against GFP slices were incubated with the rabbit anti-GFP antibody (1:200, Life Technologies, Germany) overnight at 4°C, followed by incubation with the donkey anti-rabbit Cy2-coupled secondary antibody (1:200, Dianova, Germany). Sections were mounted with Fluoroshield with DAPI containing mounting media (Sigma-Aldrich, Germany).

Up to 600 tiles per slice were acquired by a motorized Axio-Observer Z1 microscope equipped with an ApoTome optical sectioning unit (Carl Zeiss, Jena, Germany). For quantification of the GFP-fluorescent areas, computational analysis was conducted using the Cell Profiler software (35). Threshold levels were determined on mock-treated tumors. Autofluorescent tumor areas were excluded by applying a smoothing filter. For each tumor the total identified GFP-positive area was calculated by the Cell Profiler software.

## Results

### Generation and Basic Characterization of CD133-Targeted OVs

**Figure 1:**
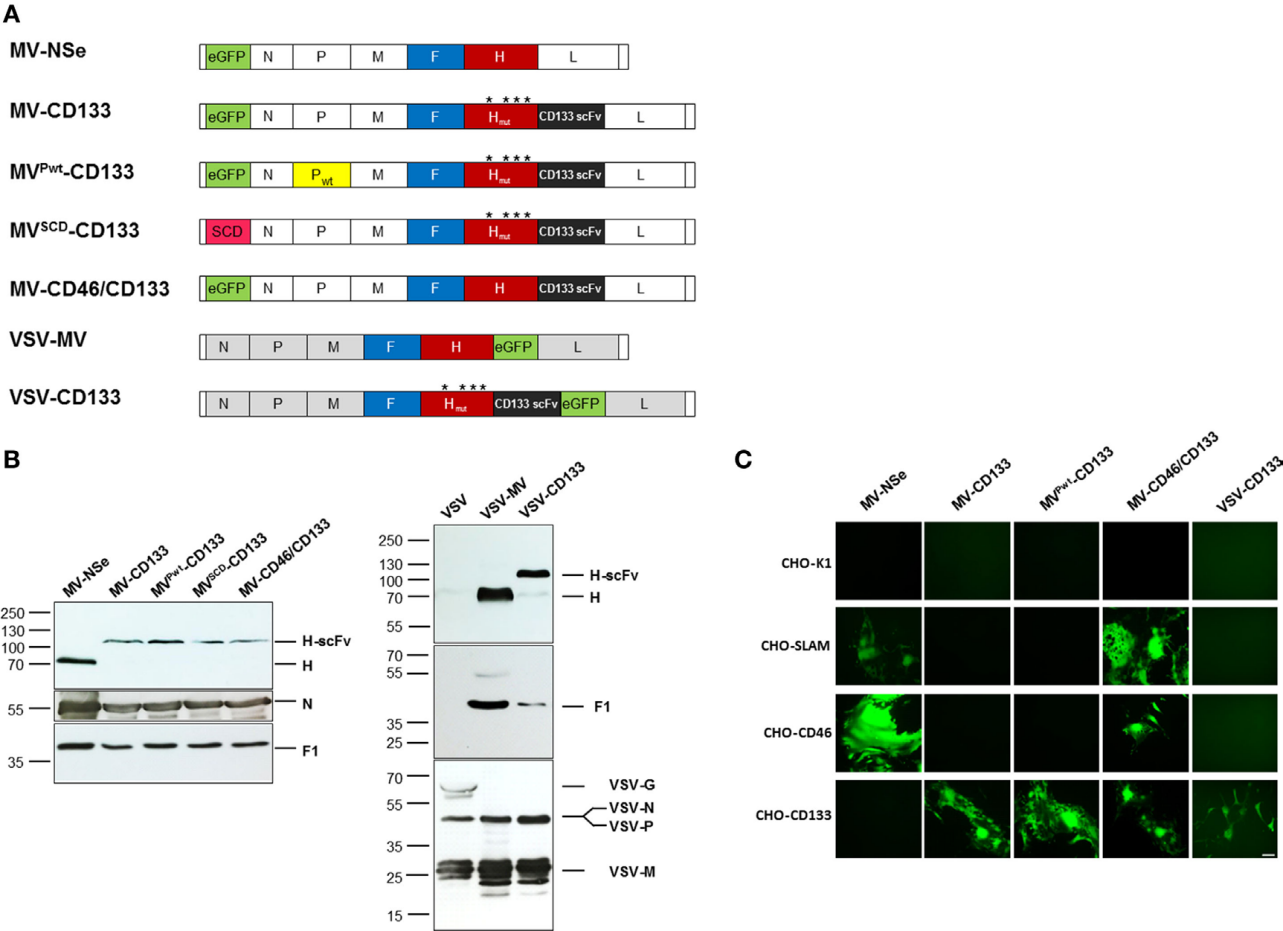
Generation of CD133-targeted oncolytic viruses. **(A)** Schematic overview on the genomic organization of the OVs used in this study. Point mutations in H protein introduced to ablate natural receptor usage are indicated by asterisks. **(B)** Immunoblot showing the incorporation of measles virus glycoproteins into recombinant measles virus (MV) and vesicular stomatitis virus (VSV) particles. Supernatants of Vero-αHis cells infected with the indicated viruses were denatured followed by fractionation by SDS-PAGE. Viral glycoproteins were detected with polyclonal antibodies directed against the indicated proteins. The parental MV-NSe, respectively, VSV and VSV-MV served as unmodified controls. N blots were used as loading control in both cases. **(C)** A panel of receptor-transgenic Chinese hamster ovary (CHO) cells (as indicated) was infected with MV-NSe, MV-CD133, MV^Pwt^-CD133, MV-CD46/CD133, or VSV-CD133 at an MOI of 0.03. CHO-K1 served as receptor-negative cell line. GFP-fluorescent images were taken 72 h postinfection; Scale bar, 200 µm.

To improve the antitumoral potency of the CD133-targeted oncolytic virus MV-CD133, we followed several strategies. First, we exchanged the P gene against that of a wild-type MV strain resulting in MV^Pwt^-CD133 (19). Alternatively, the GFP reporter gene was exchanged against the suicide gene super cytosine deaminase (SCD), which converts the prodrug 5-fluorocytosine (5-FC) into 5-fluorouracil (5-FU) resulting in MV^SCD^-CD133 (36). To extend the tropism of MV-CD133 from CD133-positive cells also to CD133-negative tumor cells, MV-CD46/CD133 was generated, in which the CD133-specific scFv was fused to unmodified H protein. Finally, we transferred the CD133-targeting strategy to VSV by exchanging the VSV glycoprotein gene against the CD133-targeted MV-H and the MV-F genes resulting in VSV-CD133 (Figure 1A).

After rescuing the panel of oncolytic viruses, we first assessed the protein composition of each virus type by Western blot analyses. All MV-derived viruses contained comparable amounts of F1 and N proteins and showed the expected shift in the electrophoretic mobility of the H-scFv fusion proteins compared to unmodified H present in MV-Nse (Figure 1B). The protein composition of VSV-CD133 was compared to those of VSV-MV and VSV. Whereas the glycoprotein G was detected in the lane loaded with VSV, this signal was absent in lanes loaded with VSV-MV or VSV-CD133. Instead, the latter viruses showed the measles H and F1 proteins. There was no difference in the amounts of the VSV-N, P and M proteins detectable between the VSV-MV chimeras and VSV (Figure 1B).

To address usage of CD133 as entry receptors, CHO cells transgenic for the natural entry MV receptors CD46 or SLAM, or the target receptor CD133 were infected with MV-NSe, MV-CD133, MV^Pwt^-CD133, MV-CD46/CD133, or VSV-CD133 at low multiplicity of infection. The parental CHO-K1 cell line served as negative control. As shown in Figure 1C, MV-NSe infected only those cell lines expressing CD46 or SLAM. While the CD133-targeted MV and VSV viruses exclusively infected CHO-CD133 cells, MV-CD46/CD133 was able to infect CD46- and CD133-positive CHO cells as well as SLAM-positive cells. Syncytia formation was comparably strong between all MVs. Lysis was most pronounced in cells infected with VSV-CD133. The parental cell line CHO-K1 showed no green fluorescence.

These results revealed that the cell tropism of the oncolytic viruses was selective and restricted to cell lines expressing the targeted surface receptors.

### CD133-Targeted OVs Do Not Impair the Differentiation Potential of Human HSCs

Oncolytic viruses not only must be effective but also have to be safe. In particular, this means that healthy cells endowed with the target receptor should be spared by the targeted oncolytic virus. Besides tumor-initiating cells, CD133 is also a marker for early progenitors of the hematopoietic system. Infection of these cells by the CD133-targeted viruses could result in tremendous side effects such as myelosuppression. To determine the proliferative and differentiation capacity of hematopoietic progenitors incubated with the OVs generated here, we performed colony-forming assays (CFAs). Flow cytometric analyses of CD34-positive human hematopoietic cells confirmed high cell surface expression levels of both, CD46 and CD133 (Figure S1 in Supplementary Material). The cells were plated into semisolid methylcellulose medium supplemented with cytokines and growth factors enabling the cells to proliferate and differentiate to produce clonal clusters of maturing cells of the hematopoietic lineage. Myeloid progenitors and committed progenitors of the erythroid, monocyte, and granulocyte lineages could then be enumerated and identified according to their morphology.

**Figure 2:**
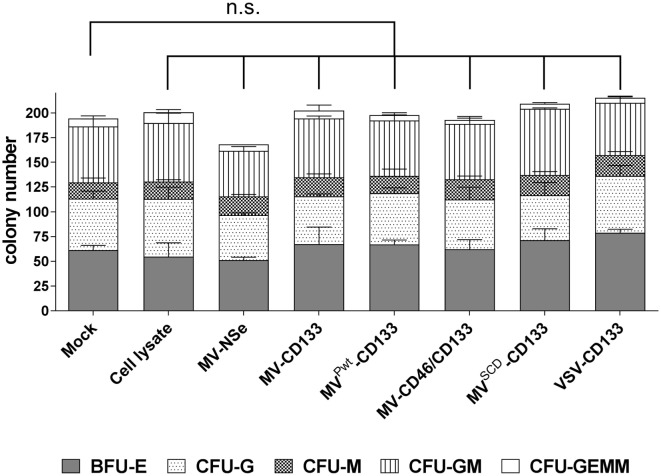
Hematopoietic stem cell properties are not impaired by infection. A colony-forming assay was performed with human CD34-positive cells purified from G-CSF mobilized peripheral blood that were incubated with the indicated viruses (MOI of 1), lysate from uninfected Vero-αHis cells, or PBS (mock). After 11 days, the number of colonies derived from erythroid and myeloid progenitors was determined by light microscopy. The proportion of the respective progenitors is shown in relation to the total colonies. Mean distribution ± SD of all colonies derived from three technical replicates is shown as a bar. The statistical analysis was carried out by a descriptive-explorative data analysis. Differences between treatment groups and the mock control group were not significant according to one-way ANOVA followed by Sidak’s multiple comparison test: *P* = 0.9993 (cell lysate), *P* = 0.4103 (MV-Nse), *P* > 0.9969 (MV-CD133), *P* > 0.9999 (MV^Pwt^-CD133), *P* > 0.9999 (MV-CD46/CD133), *P* = 0.9036 (MV^SCD^-CD133), and *P* = 0.6528 (VSV-CD133). BFU-E, burst-forming unit erythroid; CFU-G, colony-forming unit-granulocyte; CFU-M, colony-forming unit-macrophage; CFU-GM, colony-forming unit-granulocyte, macrophage; CFU-GEMM, colony-forming unit-granulocyte, erythroid, macrophage, megakaryocyte; n.s., not significant; VSV, vesicular stomatitis virus; MV, measles virus.

CD34-positive cells were subjected to the CFA 3 days postinfection with an MOI = 1 of each virus type. After 11 days, colonies derived from maturing hematopoietic progenitor cells were identified and quantified by light microscopy. In each sample, hematopoietic progeny of all lineages covered by this assay were detected. Colony numbers in total and subparts of all treatment groups were not decreased compared to mock-treated cells (Figure 2). However, a slight decrease of the total colony number of HSCs, which were infected with MV-NSe, was found. The difference in the proliferation and differentiation of cells derived from mock, cell lysate and virus-infected cells was not significant (Figure 2). Furthermore, none of the colonies contained any GFP-positive cells. The results demonstrate that the CD133-targeted viruses investigated here do not impair the hematopoietic capabilities of CD34-positive cells.

### Infection and Killing of Tumor Cells

**Figure 3:**
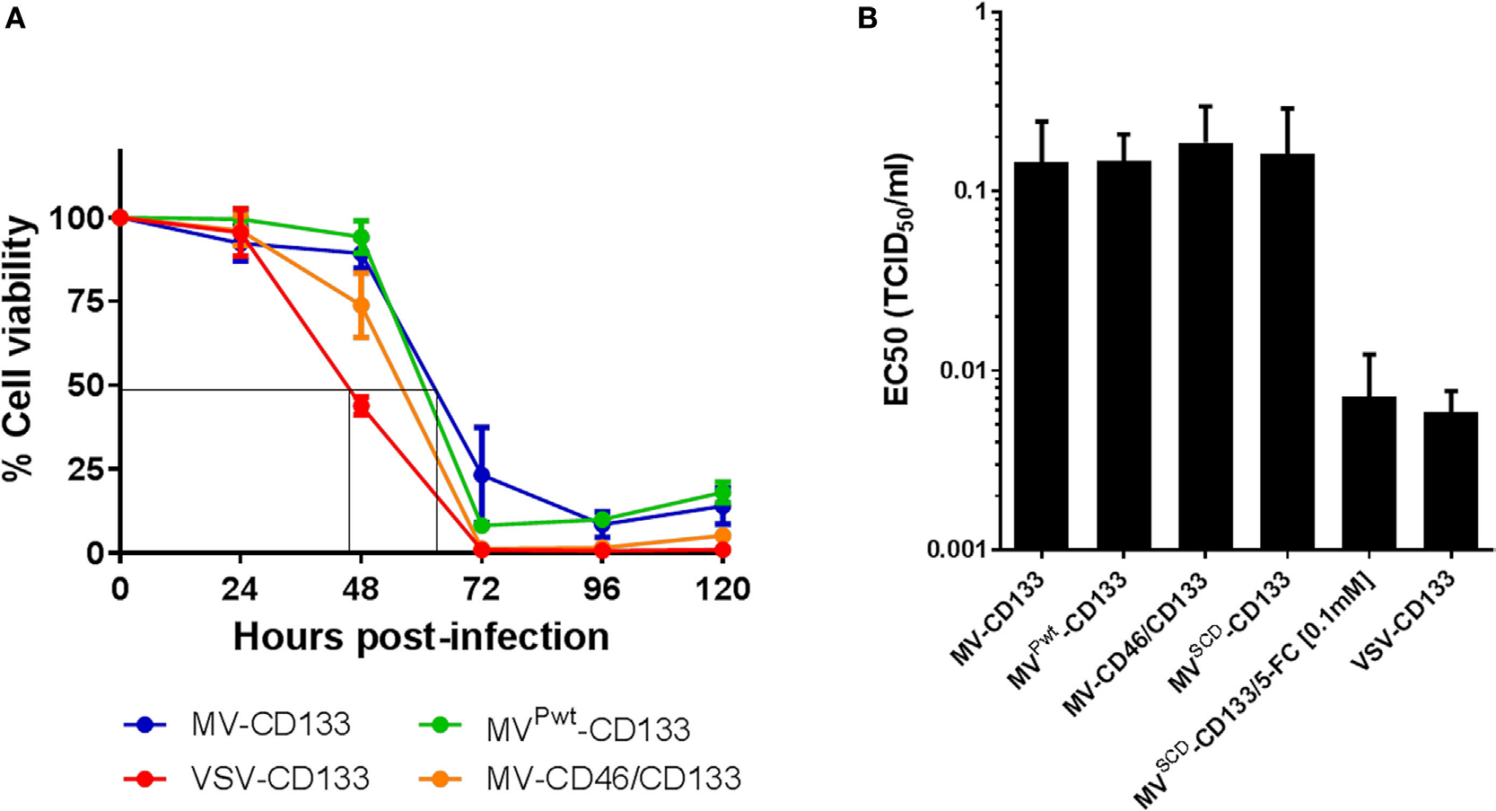
Comparison of the *in vitro* killing performance of oncolytic viruses on Huh7 cells. Cells were infected with the indicated viruses, and cell viability was measured every 24 h postinfection by WST assays. **(A)** Cell viability after infection at an MOI of 1 at the indicated time points. Depicted is the percentage of living cells in relation to mock-infected control culture, which was set to 100%. The results are an average of two biological and four technical replicates. **(B)** EC_50_ values of the indicated viruses determined at 72 h postinfection relative to untreated controls. The results are an average of three biological and four technical replicates.

Next, the oncolytic activity of the newly established viruses was analyzed in cell killing assays, at first using the CD133-positive hepatocellular carcinoma cell line HuH7. In killing kinetics using an MOI = 1, MV^Pwt^-CD133, MV-CD46/CD133, or VSV-CD133 killed nearly 100% of the cells within 72 hpi, while MV-CD133 lagged slightly behind (Figure 3A). Cell viability was reduced to less than 50% before 48 hpi in VSV-CD133 infected cells. VSV-CD133 was thus at least 15 h faster than the MVs. To further quantify the dose dependency of the killing capabilities, tumor cells were infected with different MOIs to determine the effective concentrations (EC_50_) required to reduce the cell viability to 50% relative to untreated controls. Infection with all viruses resulted in substantial dose-dependent killing of HuH7 cells, with VSV-CD133 and MV^SCD^-CD133 (in combination with 5-FC) being substantially more active than the other viruses at low MOIs (Figure S1 in Supplementary Material). Accordingly, the EC_50_ of VSV-CD133 and MV^SCD^-CD133 (+5-FC) was reached at 31-fold and 26-fold, respectively, less infectious virus particles than for the other viruses (Figure 3B).

**Figure 4:**
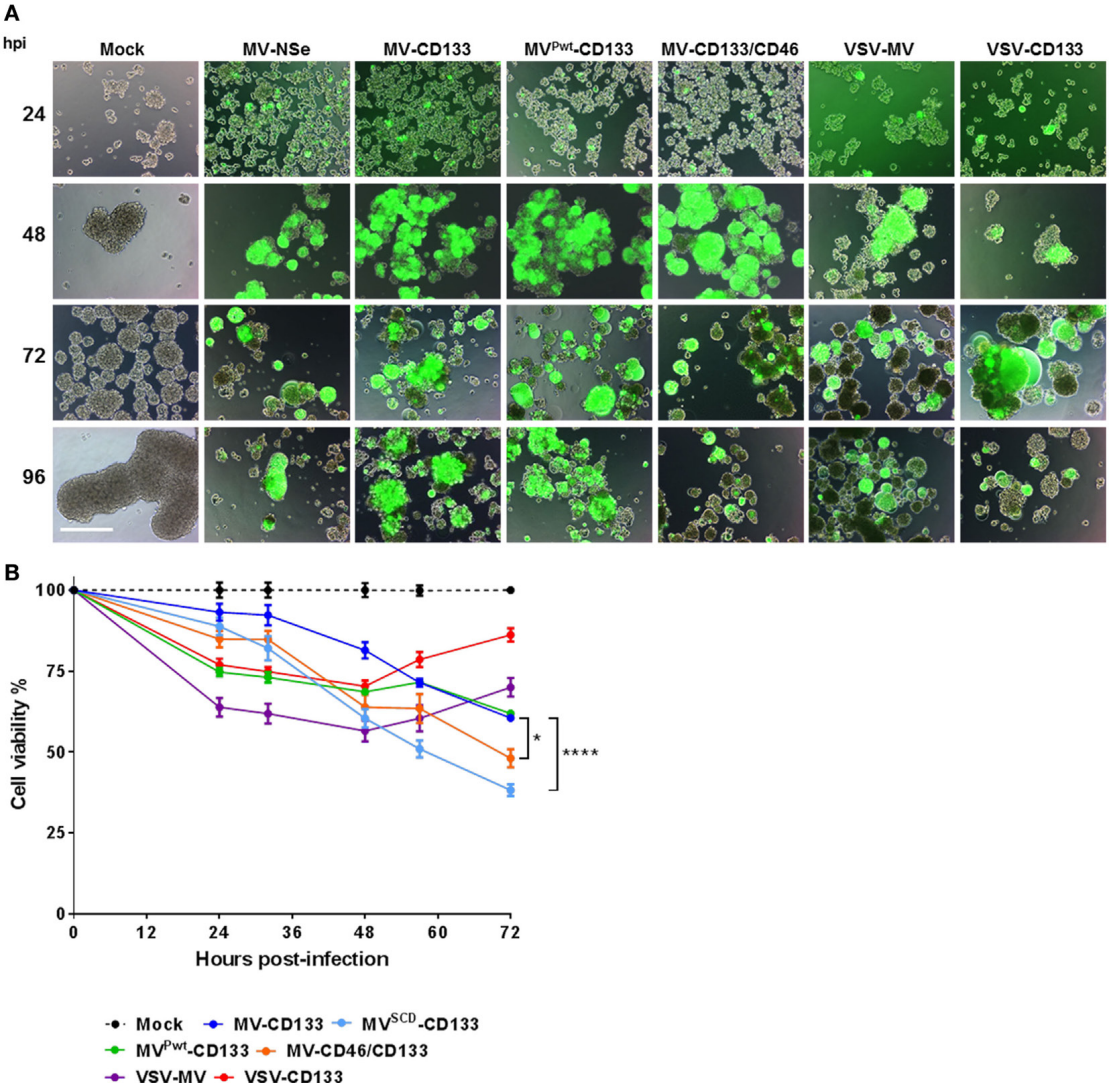
Infection and killing of primary glioma spheres. Single cell suspensions of NCH644 cells were infected with the indicated viruses at an MOI of 1, respectively. **(A)** Cells were monitored microscopically for GFP expression up to 96 h postinfection. Scale bar, 500 µm. **(B)** Cell viability was determined using the RealTime-Glo MT Cell Viability assay twice a day for 72 h after virus addition. 1 mM 5-FC was added to MV^SCD^-CD133-infected cells only, at time point 21 hpi. Average values of three independent killing assays are shown. One-way ANOVA with Dunnett’s multiple comparison test, *P < 0.05; ****P < 0.0001.

To determine the oncolytic activity of the viruses against primary tumor cells, we assessed the infection of the primary glioma cells NCH644, which are about 90% positive for CD133 and form spheres under serum-free conditions (27). Tumor cells were infected as single cell suspension at an MOI of 1. Green fluorescent cells became detectable 24 h postinfection with strongest signals for MV^Pwt^-CD133 and MV-CD46/CD133. Cytopathic effects were most prominent in cells infected with MV-CD46/CD133. However, all viruses were able to infect glioma tumor spheres and to induce syncytia formation (Figure 4A). To quantify cell killing we monitored the cells over time. All viruses killed more cells with increasing dose (Figure S2 in Supplementary Material). Over time, however, a steady reduction in cell numbers was observed for the MV-derived viruses but not for VSV-CD133 and VSV-MV (Figure S2 in Supplementary Material). In fact, cell killing reached its maximum by 24 h with these viruses. From then on cells started to propagate again (Figure S2 in Supplementary Material; Figure 4B). Supernatant from MV-CD133 and VSV-CD133 infected cells collected at time points 12, 24, 48, and 72 postinfection did not contain any evidence for the presence of IFNα in any of the samples as tested by ELISA with a sensitivity of 6.25 pg/ml (*N* = 3 biological replicates). Among the MVs, cell killing was most efficient with MV-CD46/CD133 and also with MV^SCD^-CD133, after having identified the optimal prodrug concentration (Figure S3 in Supplementary Material; Figure 4B). These viruses were significantly more efficient in killing glioma tumor spheres than MV-CD133 or MV^Pwt^-CD133 at 72 hpi.

### MV-CD46/CD133-Treated Mice Show a Survival Benefit in an Orthotopic Glioma Model

**Figure 5:**
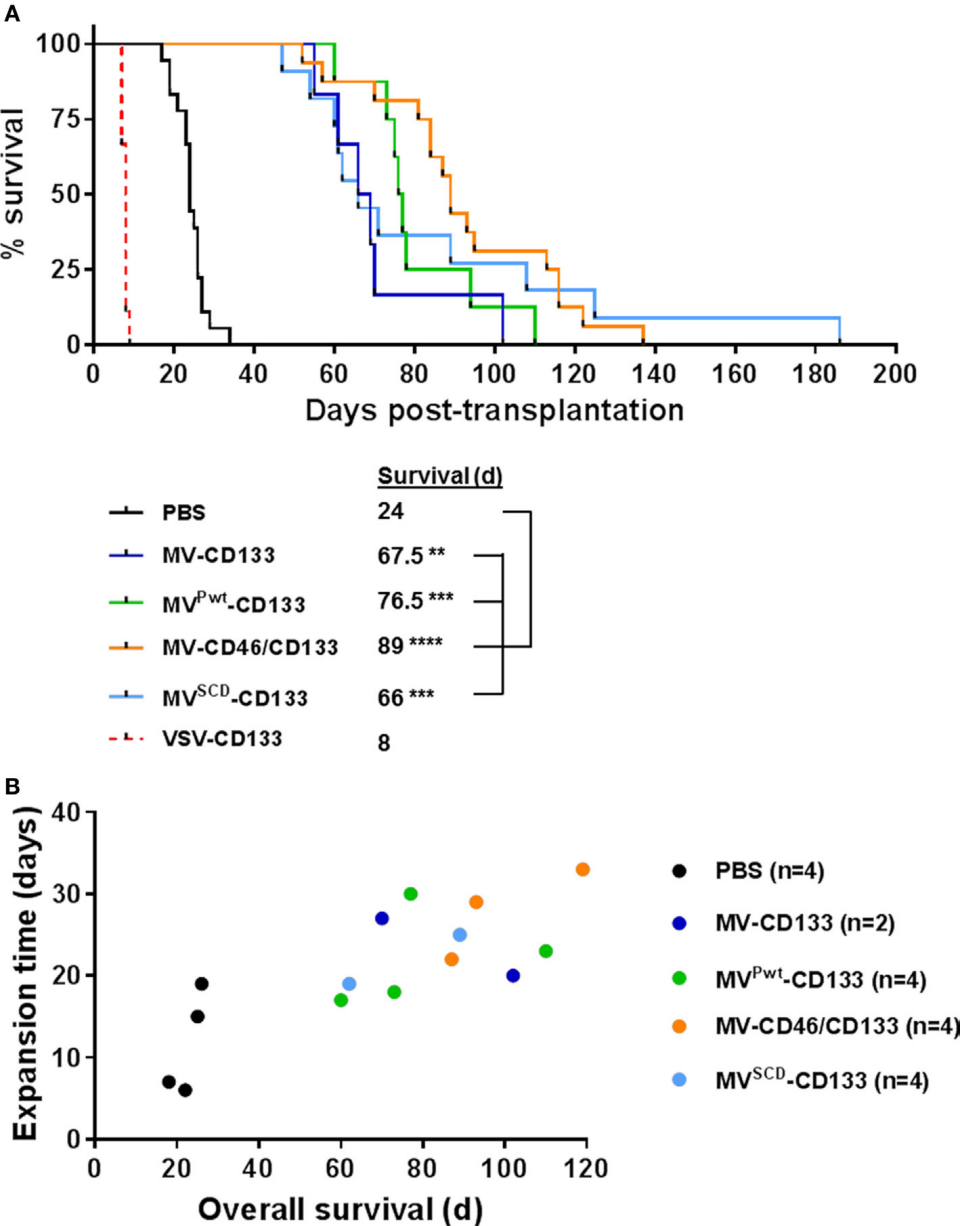
Intracranial implantation of preinfected glioma spheres. Primary glioma spheres were infected with the indicated viruses at an MOI of 0.5 followed by stereotactically assisted implantation into the corpus striatum of NOD-SCID mice 16 h later. Mice having received MV^SCD^-CD133-infected cells received 5-FC (200 mg/kg body weight) twice per day intraperitoneally initiated 3 day postimplantation for four consecutive days. **(A)** Health status and body weight were monitored daily. Mice were sacrificed with onset of neurological symptoms and/or weight loss of more than 20%. Based on the defined end points, a Kaplan–Meier survival curve was generated. Comparison of the mean survival periods between the mock control and the treatment groups was conducted by a log-rank test followed by a Bonferroni correction for multiple comparisons. Log-rank test, **P < 0.01; ***P < 0.001; ****P < 0.0001; PBS, *n* = 18; measles virus (MV)-CD133, *n* = 6; MV^Pwt^-CD133, *n* = 8; MV-CD46/CD133, *n* = 16; MV^SCD^-CD133, *n* = 11; vesicular stomatitis virus (VSV)-CD133, *n* = 9. **(B)** At the experimental endpoint, tumors were explanted and identical numbers of cells cultivated. The time required to expand to a cell count of 1 × 10^6^ was plotted against the median survival time of the respective animal.

In a next step, we intracranially implanted NCH644 glioma spheres infected shortly before injection and followed survival of the mice. With the exception of VSV-CD133, all mice treated with oncolytic viruses survived substantially longer than untreated mice (Figure 5A). MV-CD46/CD133 showed a tendency toward being most effective, since mice treated with this oncolytic agent survived on average longer than all others (median survival = 89 days). Interestingly, some individuals from the MV^SCD^-CD133-treated group survived especially long (more than 100 days). However, the median survival of this group was below that of the MV-CD46/CD133-treated group. Unexpectedly, all mice treated with VSV-CD133 had to be sacrificed much earlier than the control group (at day 8 post transplantation) due to severe peracute neurologic symptoms such as ataxia, tremor, or apathy.

To analyze the remaining stemness properties of the tumor cells present in the brains of sacrificed mice, we explanted some of the tumors and assayed the cells for sphere formation and proliferation. All samples derived from mock (*n* = 4), MV-CD133 (*n* = 2), and MV^Pwt^-CD133 (*n* = 4) treated animals were able to produce spheres *ex vivo*, whereas this was the case for only three out of four tumor specimens of MV-CD46/CD133-treated animals (*n* = 4) and only half, i.e., two, of the tumors treated with MV^SCD^-CD133 (*n* = 4). Moreover, there was a tendency for tumor cells that were removed from animals with longer survival times to require longer cultivation times to reach a defined cell number (Figure 5B). There were no signs for GFP expression in these cells that could point to a persistent infection with virus.

### Direct Intracranial Application of the Oncolytic Viruses

**Figure 6:**
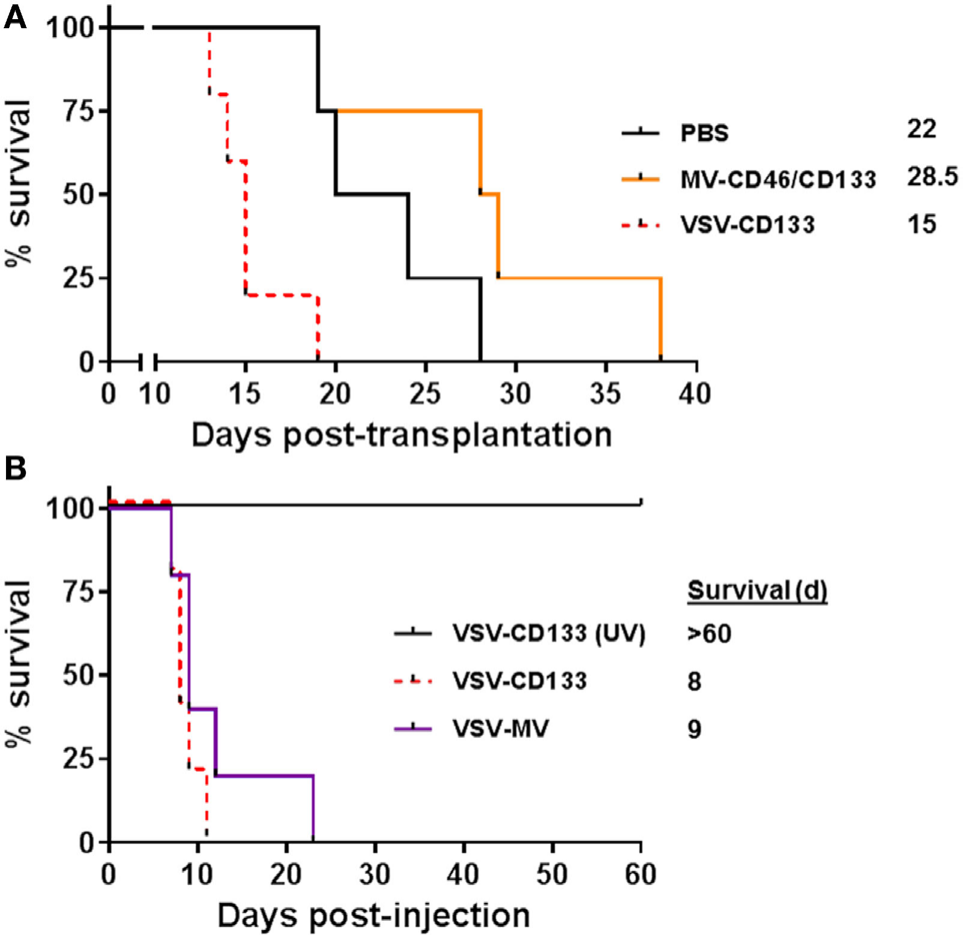
Intracranial injection of oncolytic viruses. **(A)** 1 × 10^5^ primary glioma sphere cells were stereotactically implanted into the corpus striatum of NOD-SCID mice. After 5 days, 2 × 10^5^ TCID_50_ of the indicated viruses in 5 µl PBS or PBS were stereotactically injected into the same coordinates. Health status and body weight were monitored daily. Mice were sacrificed with onset of neurological symptoms and/or loss of weight by more than 20%. Based on the defined end points Kaplan–Meier survival plots were generated. PBS, *n* = 4; measles virus (MV)-CD46/CD133, *n* = 4; vesicular stomatitis virus (VSV)-CD133, *n* = 5. **(B)** 2 × 10^5^ TCID_50_ of VSV-CD133, UV-inactivated (UV) VSV-CD133, or VSV-MV in 5 µl PBS, respectively, were stereotactically injected into the corpus striatum of NOD-SCID mice. Health status and body weight were monitored daily. Mice were sacrificed with onset of neurological symptoms and/or weight loss of more than 20%, *n* = 5.

We next evaluated the antitumoral activities of MV-CD46/CD133 and VSV-CD133, respectively, in the orthotopic glioma model in a clinically more relevant setup by direct intracranial injection of the viruses into pre-established tumors. 1 × 10^5^ NCH644 cells were implanted intracranially into NOD-SCID mice. Five days later, 2 × 10^5^ TCID_50_ of the oncolytic viruses or PBS as control were stereotactically injected through the same hole, respectively. Mice treated with MV-CD46/CD133 revealed a tendency for longer survival (median survival = 28.5 days) over mock-treated animals (Figure 6A). All VSV-CD133-treated mice developed neurological symptoms within 15 days and thus had to be sacrificed earlier than PBS treated mice (Figure 6A). To assess a potential influence of the tumor cells on the observed neurotoxicity we intracerebrally injected VSV-CD133 or UV-irradiated VSV-CD133 into tumor-free mice. As control we included VSV-MV which does not use CD133 as entry receptor. Mice injected with UV-inactivated VSV-CD133 survived up to the end point of the study without developing any symptoms. In sharp contrast, animals from both other groups came down with neurological symptoms within two weeks (Figure 6B). This adverse event must thus have been caused by combining the MV glycoproteins with VSV but not by display of the CD133-specific scFv.

### VSV-CD133 Is Superior to MV-CD133 in a Subcutaneous Xenograft Model

**Figure 7:**
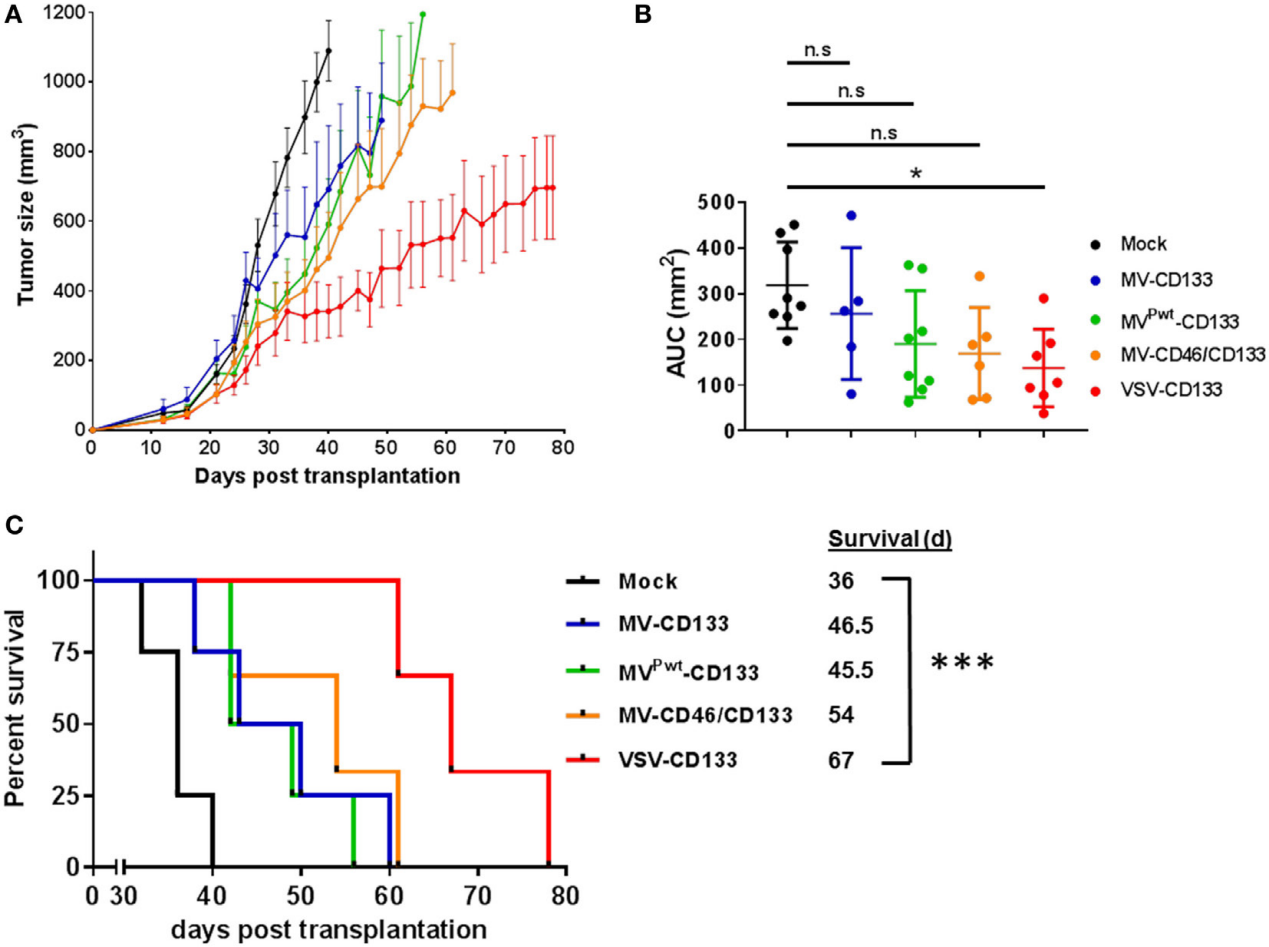
Oncolytic activities of intratumorally applied oncolytic viruses in a subcutaneous xenograft model of hepatocellular carcinoma. HuH7 cells were implanted subcutaneously into the flank of NOD/SCID mice. Tumor size was monitored using a caliper. Intratumoral treatment was initiated at an approximate tumor volume of 100 mm^3^ with four applications of 1 × 10^6^ TCID_50_ of each of the viruses on four consecutive days. Virus samples were blinded prior application, and the monitoring was carried out in a blinded fashion over the whole course of the study until sacrifice. **(A)** Tumor dimensions of each individual animal were measured twice a week. The mean tumor volume (mm^3^) of each treatment group was calculated and plotted against the course of the observation point. **(B)** Group comparisons were performed by determining the area under the curve (AUC) for each individual animal normalized against the value obtained from the last survivor of the mock group (40 days after treatment). The values were plotted as box and whiskers. One-way ANOVA with Dunnett’s multiple comparison test, *P < 0.05. **(C)** Survival data were depicted as Kaplan–Meier survival curves. Comparison between the group with the lowest median survival (MV^Pwt^-CD133) and the mock group was conducted by a log-rank test with Bonferroni adjustment for multiple comparisons. Mock, *n* = 4; measles virus (MV)-CD133, *n* = 4; MV^Pwt^-CD133, *n* = 4; MV-CD46/CD133, *n* = 3; vesicular stomatitis virus (VSV)-CD133, *n* = 3. ***P < 0.001.

To test the oncolytic performance of the CD133-targeted viruses toward HCC, we established subcutaneous xenograft tumors in NOD/SCID mice using HuH7 cells. Initially, we intratumorally injected 1 × 10^6^ TCID_50_ of each virus in four administrations, respectively, once the tumor volume had reached 100 mm^3^. Compared to mock-treated mice, all viruses reduced tumor growth, with VSV-CD133 being most effective (Figure 7A). Considering the area under the tumor growth curve (AUC) on day 40, by which all mice of the mock group had been sacrificed, the reduction in tumor growth was significant just for the VSV-CD133 treatment cohort (Figure 7B). This was also reflected by the Kaplan–Meier survival curves, which showed that treatment with VSV-CD133 resulted in the most pronounced survival benefit (Figure 7C).

**Figure 8:**
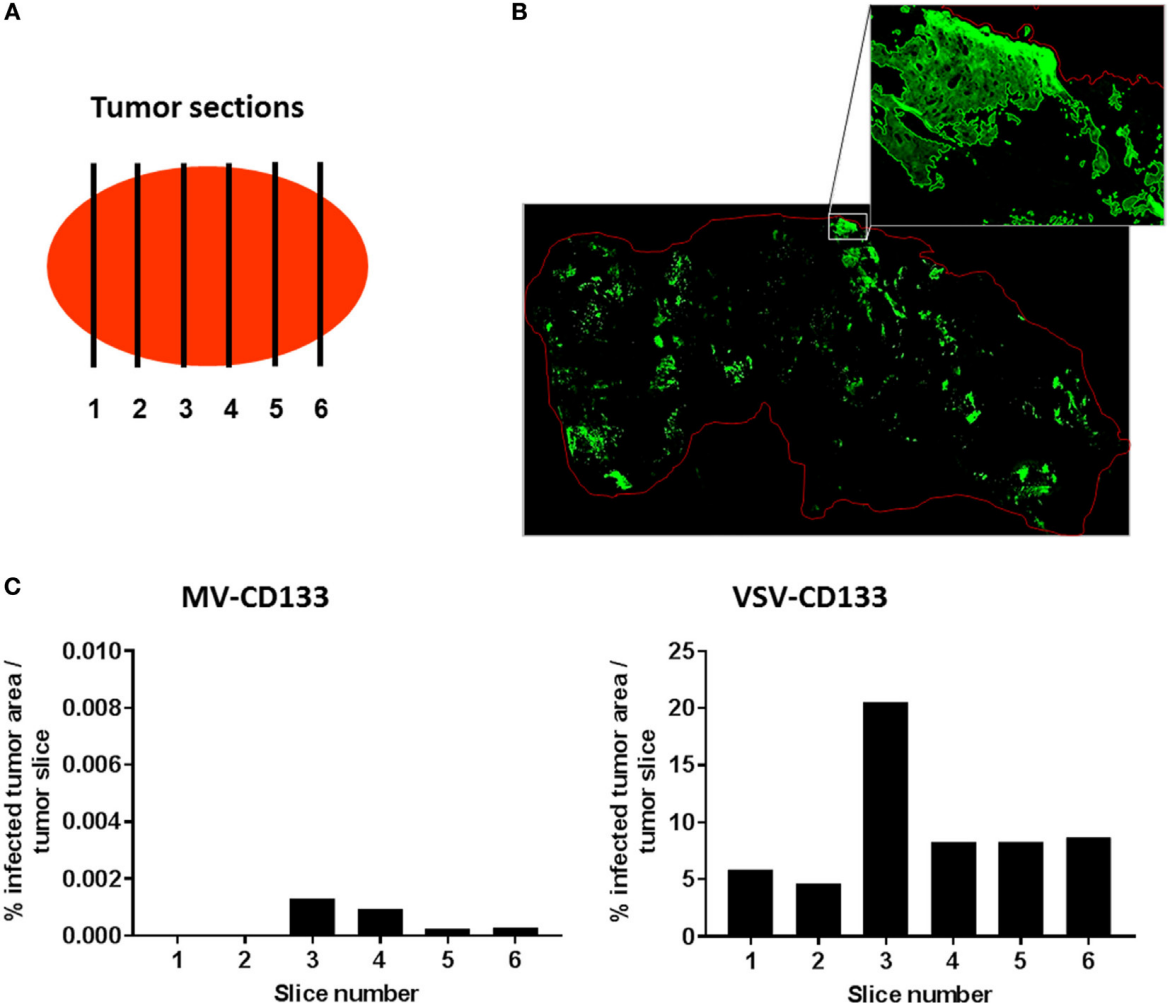
Quantification of infected areas in explanted tumors. Cryosections of explanted tumors from vesicular stomatitis virus (VSV)-CD133 or measles virus (MV)-CD133-treated mice shown in Figure 7 were immunostained against GFP to visualize virus infection centers. (**A**) Schematic illustration of the distribution of sections chosen to cover the complete tumor. (**B**) Representative overview image of a cross-section of a tumor from mice injected with VSV-CD133 (top) or MV-CD133 (bottom). Shown is a composite image that was generated by tile-by-tile acquisition using the AxioVision MosaiX software. The zoom-in represents the output image of one tile reconstructed by the CellProfiler software. The red line indicates the tumor border. (**C**) Bar graph showing the quantification of infected tumor area per slice of one tumor. The percentage of infected tumor area within the whole tumor area is plotted for each tumor site indicated in panel (**A**).

To quantify the intratumoral spread of VSV-CD133 or MV-CD133 we performed GFP immunostaining of a series of cryo-slices. Slices were prepared throughout two tumors of each virus-treatment group and of the mock-treated group as control by covering six different sites within each tumor, respectively (Figure 8A). There were no fluorescence signals detectable in slices from the mock group. For quantification of GFP-fluorescent areas, tile-by-tile acquisition of tumor cross-sections was performed using the AxioVision MosaiX software. In all viewed sections we observed a tremendous difference between tumors from VSV-CD133 and MV-CD133-treated mice (Figure 8B). Each slice from VSV-CD133-treated mice contained many GFP-positive spots, whereas more than half of the slices from MV-CD133-treated animals were GFP-negative. Taken all slices together, VSV-CD133 infected a 2 × 10^4^ times larger area than MV-CD133 (Figure 8C).

**Figure 9:**
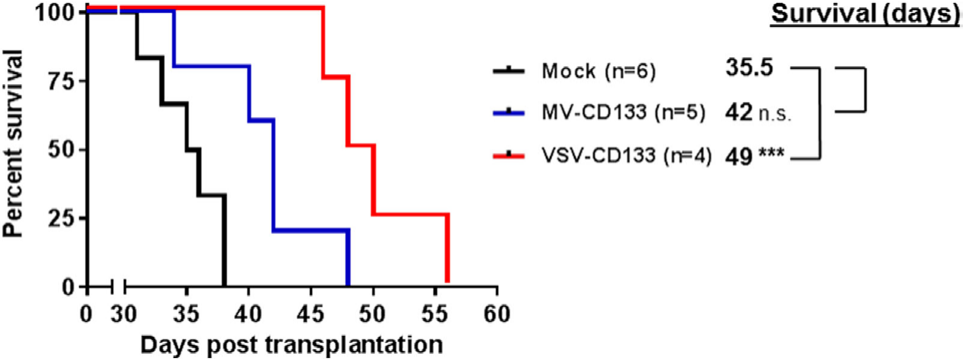
Oncolytic activity of intravenously administered viruses in a subcutaneous xenograft model of hepatocellular carcinoma. HuH7 cells were implanted subcutaneously into the right flank of NOD/SCID mice. Intravenous injection of 1 × 10^6^ TCID_50_ of vesicular stomatitis virus (VSV)-CD133 (*n* = 4) or measles virus (MV)-CD133 (*n* = 5), respectively, or PBS as control (mock; *n* = 6) was initiated at an approximate tumor volume of 100 mm^3^ and performed three times every second day. Survival data were depicted as Kaplan–Meier survival curves. Comparisons of both treatment groups with the mock group were conducted by a log-rank test with Bonferroni adjustment for multiple comparisons. ***P < 0.001.

After the intratumoral treatment setup, we performed a clinically more relevant approach by injecting VSV-CD133 or MV-CD133 intravenously into mice bearing subcutaneous HuH7 tumors. In this setting, both viruses prolonged survival, but significance was reached for the VSV-CD133 treatment only (Figure 9).

## Discussion

CD133-positive tumor cells represent a prime target for the development of novel cancer therapeutics. Conventional drugs as well as immunotherapies are under development including CD133-specific antibodies coupled to toxins, CD133-specific BITEs, or CD133-targeted chimeric antigen receptors (37–40). We have previously added oncolytic MV engineered to use CD133 as receptor for cell entry to this list (27). In the current study we aimed at enhancing the oncolytic activity of MV-CD133 through arming either with the suicide gene SCD or the P gene from wild-type MV, through extending receptor usage from CD133-only to the natural MV receptors, and by transferring the CD133-targeted MV envelope to VSV. The later approach resulted in VSV-CD133, the by now second example of a fully replication-competent, receptor-targeted VSV. VSV-CD133 and all the other newly generated viruses entered cells *via* CD133 and were highly selective for CD133-positive cells with the exception of MV-CD46/CD133, which, as expected, also infected CD133-negative but MV-receptor positive cells.

We performed a careful side-by-side comparison of the whole panel of viruses toward their capability to lyse the hepatocellular carcinoma derived tumor cell line HuH7 or glioma derived tumor sphere cells, both *ex vivo* and *in vivo*. Since all viruses use the same surface protein for entry, the comparison was unbiased through usage of different receptors as it is usually the case when oncolytic viruses are being compared. Injection of the viruses resulted in reduced tumor growth, but not a complete elimination of tumor cells. Complete eliminations of tumors are in general rarely seen in preclinical models after single round treatments with oncolytic viruses, especially in immunodeficient mice, where an antitumoral immune response triggered by the virus infection cannot add to the oncolytic effect. Reasons for this can, e.g., be inaccessibility of the tumor cells, low virus dose or resistance against the virus in a subset of tumor cells. While in our system the latter could have occurred through loss of CD133 expression, also MV-CD46/CD133 with its expanded tropism was unable to completely clear tumors in the given setting, arguing against a major contribution of CD133 downregulation. Future experiments assessing multiple dosing cycles, the oncolytic activity in immunocompetent animal models and infection experiments with tumor cells re-isolated from treated animals will clarify this.

Interestingly, we identified different virus types as best option for each tumor type. On HuH7, VSV-CD133 was by far most efficient in infection and tumor cell lysis. This was also the case *in vivo* in subcutaneous HuH7 tumors. After intratumoral injection VSV-CD133 reached basically all parts of the tumor tissue thus covering an area more than 10^4^ times larger than that reached by MV-CD133 during the same time period. This impressive capacity in spreading through tumor tissue, which was reflected by our observation that VSV-CD133 was also efficacious when injected intravenously, can be regarded as important property to reach and destroy CSCs in a clinical situation of HCC, where CD133-positive CSCs make up a low percentage of all cells present in tumor tissue (4, 41). In such a situation, it may be worth considering to also extend the receptor usage of VSV-CD133 to CD46, since our data show that MV-CD46/CD133 was superior over MV-CD133 in both tumor models. Being capable of using both these surface proteins as entry receptors should increase the likelihood to reach and infect rare CSCs.

The situation was different on glioma tumor spheres. Here, MV-CD46/CD133 and MV^SCD^-CD133 turned out to be the most promising viruses. VSV-CD133 was more effective and more rapid in infection and cell killing *in vitro* only within the first 24 h, and even when used at an MOI of 10 was unable to kill more than about 60% of the cells. In fact, 2–3 days after infection tumor spheres started expanding again. The MV-derived viruses in contrast caused a continuous decrease in cell number, most efficiently with MV^SCD^-CD133 and MV-CD46/CD133. It is rather unlikely that this was due to loss of CD133 expression. CD133-negative cells can indeed be killed by MV-CD46/CD133 or MV^SCD^-CD133 because of CD46 receptor usage or the SCD-mediated bystander activity. However, NCH644 tumor spheres contain more than 90% CD133-positive cells and infection with VSV-MV, relying on CD46 for cell entry, showed the same recovery of the cells observed for VSV-CD133. It is therefore more likely that the cells became resistant through a postentry mechanism. Since VSV is known to be highly sensitive toward the IFN-mediated innate immune response, an intact type-I IFN response in some of the cells could be an explanation. However, absence of IFN-α in the supernatant of the infected cells rather argues for an alternative mechanism. This is further supported by the absence of any oncolytic advantage of the Pwt-armed MV^Pwt^-CD133, which was expected to be especially pronounced in presence of a type-I IFN response. The resistance of NCH644 cells must therefore be the subject of further studies. Despite the negative results obtained for MV^Pwt^-CD133 in our study, preclinical testing of this virus may still be worthwhile on other tumor types, especially those with an active type-I IFN system.

A postentry block triggered by the IFN system is most likely also the reason for the unimpaired differentiation of HSCs infected with the panel of viruses generated in our study into the hematopoietic lineages. Independently from the molecular mechanism, this is an important result with respect to the safety of CD133-targeted viruses. We had previously shown that HSCs appear to be protected from infection with MV-CD133 but did not exclude any influence on their differentiation capability (27).

An unexpected outcome of our study was the severe neurotoxicity exerted by VSV-CD133. All mice intracerebrally transplanted with glioma tumor spheres infected with VSV-CD133 came down with neurological symptoms within 10 days. VSV is indeed known for its distinct neurotoxicity. However, strategies have been developed to attenuate the virus to become applicable as oncolytic agent, and first clinical trials have been initiated (42). Our observation is especially astonishing, since the glycoprotein G had been identified as main neurotoxic component and mutating or exchanging it against envelope proteins of other viruses usually abolishes neurotoxicity (42–44). Neurotoxicity of vaccine strain-derived oncolytic MV has by now only been observed in CD46 transgenic and IFN receptor deficient mice after intracerebral injection (24, 45), while intracerebral injections into non-human primates did not reveal any signs of neurotoxicity (46).

The NOD/SCID mice we applied here do neither express primate CD46 that can be utilized by MV for cell entry, nor do they have a defect in innate defense although being B and T cell deficient. Moreover, VSV-CD133 is deficient in natural MV-receptor recognition and the displayed CD133-specific scFv only recognizes human CD133. Indeed, when we assessed VSV-MV, a non-targeted virus, in which VSV-G had been replaced by MV-F/H, the same extent of neurotoxicity was observed. Notably, there were no tumor cells present in this setting, thus excluding virus burst from preinfected spheres as potential trigger for the fatal neurological signs. First hints for neurotoxicity of VSV-MV were published by Ayala-Breton et al. (47). Here, however, virus was injected intravenously and resulted in neurotoxicity only when mice expressed human CD46 (47). Since we observed neurotoxicity in mice neither expressing human CD46 nor any of the other known MV receptors in their brains, neurotoxic infection must have been mediated either by an as of yet unidentified neuronal “receptor X” contacted by H (48), or a membrane fusion process that occurred independently from H protein receptor contact. Such a process has recently been suggested for F proteins found in AIDS patients suffering from MV-induced encephalitis (49). These F proteins, like those found in SSPE patients, however, carried mutations that enhanced their fusogenic activity (50). Since VSV-MV encodes the original F protein sequence, fusogenicity in this setting would have to be enhanced through being placed in the context of VSV. Indeed, absence of the MV matrix protein is known to result in enhanced cell-to-cell fusion activity of MV glycoprotein complexes (51) In addition, the much faster replication machinery and the apoptosis-inducing VSV matrix protein (M) likely contributed to the neurotoxicity observed with the VSV-MV chimeras.

With respect to translation into clinical applications, our data further underline that careful toxicity testing of chimeric VSV-MV viruses is an essential requirement for any new type of chimeric virus. For VSV-CD133, it will have to be assessed if the neurotoxicity we observed in NOD/SCID mice will be similarly severe in immunocompetent mice. That mice with a deficient innate immune system are especially prone to neurotoxicity caused by VSVs carrying heterologous envelope glycoproteins was recently observed for chimeras containing the glycoproteins of chikungunya or influenza virus (52). IFN-deficient mice are most sensitive toward VSV, even more than NOD/SCID mice, which were safe against a VSV-Lassa virus chimera in contrast to IFN-deficient mice (53). However, certain types of VSV chimeras can also be neurotoxic in immunocompetent mice as recently observed for VSVs carrying the Nipah virus glycoproteins (52). It is currently impossible to predict which combination may be crucial. Experimental testing is therefore unavoidable.

In this context, it is also important to stress that we did not see any signs of toxicity after systemic injection of VSV-CD133. The impressive spreading of VSV-CD133 in tumor tissue and the significant prolongation of survival after systemic administration therefore warrant further testing of VSV-CD133 toward applications in cancers of the gastrointestinal tract with CD133-positive CSCs being involved. Enhancing the safety of VSV-CD133 can for example be achieved by replacing its M gene by the MΔ51 variant (42). A negative outcome of a careful neurotoxicity testing provided, hepatocellular or pancreatic cancer could then be prime candidates (4).

With respect to glioblastoma, it will be worth further exploring the therapeutic activities of MV^SCD^-CD133 and MV-CD46/CD133. As next step it will be important to assess the viruses on primary tumor material from patients suffering from these cancer types to find out if CSCs will be infected and potentially eliminated. Lysis of CSCs may, however, not be necessary. Only entering into CD133-positive CSCs and triggering a type-I interferon response may be sufficient to sensitize these cells for treatment by chemotherapy or radiation, or to induce their differentiation (54–56). Since vaccine strain-derived oncolytic MVs have lost at least part of their capacity to suppress IFN-responses and induce higher amounts of IFNs than wild-type strains (57), CSC-targeted MVs may be especially suited for such parallel modes of action.

## Ethics Statement

This study was carried out in accordance with the recommendations of the German animal protection law. The protocol with the reference number F107/1002 was approved by the Regierungspräsidium Darmstadt.

## Author Contributions

DK designed and performed experiments and contributed to the writing of the manuscript. JH performed experiments and S-KK provided advice. CA-B and K-WP were involved with the protocols and reagents. K-MH evaluated data. MM supplied reagents and contributed in the writing of the manuscript. AM and TA supervised work and performed experiments. CB conceived and designed the study, acquired grants, supervised work and wrote the manuscript.

## Conflict of Interest Statement

CB and TA are inventors of a patent on tumor-stem cell targeted oncolytic viruses that has been out-licensed. All other authors declare no conflict of interest.

## References

[1] Kahlert UD Mooney SM Natsumeda M Steiger H-J Maciaczyk J. Targeting cancer stem-like cells in glioblastoma and colorectal cancer through metabolic pathways. Int J Cancer (2017) 140(1):10–22. 10.1002/ijc.30259 27389307

[2] Bonnet D Dick JE. Human acute myeloid leukemia is organized as a hierarchy that originates from a primitive hematopoietic cell. Nat Med (1997) 3(7):730–7. 10.1038/nm0797-730 9212098

[3] Singh SK Hawkins C Clarke ID Squire JA Bayani J Hide T Identification of human brain tumour initiating cells. Nature (2004) 432(7015):396–401. 10.1038/nature03128 15549107

[4] Schmohl JU Vallera DA. CD133, selectively targeting the root of cancer. Toxins (Basel) (2016) 8(6):E165. 10.3390/toxins8060165 27240402 PMC4926132

[5] Irollo E Pirozzi G. CD133: to be or not to be, is this the real question? Am J Transl Res (2013) 5(6):563–81. 24093054 PMC3786264

[6] Wu B Sun C Feng F Ge M Xia L. Do relevant markers of cancer stem cells CD133 and Nestin indicate a poor prognosis in glioma patients? A systematic review and meta-analysis. J Exp Clin Cancer Res (2015) 34:44. 10.1186/s13046-015-0163-4 25967234 PMC4436020

[7] Li B McCrudden CM Yuen HF Xi X Lyu P Chan KW CD133 in brain tumor: the prognostic factor. Oncotarget (2017) 8:11144–59. 10.18632/oncotarget.14406 28055976 PMC5355253

[8] Zhang W Chen H Lv S Yang H. High CD133 expression is associated with worse prognosis in patients with glioblastoma. Mol Neurobiol (2016) 53(4):2354–60. 10.1007/s12035-015-9187-1 25983032

[9] Shin JH Lee YS Hong Y-K Kang CS. Correlation between the prognostic value and the expression of the stem cell marker CD133 and isocitrate dehydrogenase1 in glioblastomas. J Neurooncol (2013) 115(3):333–41. 10.1007/s11060-013-1234-z 24129546

[10] Zhong C Wu J-D Fang M-M Pu L-Y. Clinicopathological significance and prognostic value of the expression of the cancer stem cell marker CD133 in hepatocellular carcinoma: a meta-analysis. Tumour Biol (2015) 36(10):7623–30. 10.1007/s13277-015-3487-y 25921286

[11] Jang J-W Song Y Kim S-H Kim J-S Kim KM Choi EK CD133 confers cancer stem-like cell properties by stabilizing EGFR-AKT signaling in hepatocellular carcinoma. Cancer Lett (2017) 389:1–10. 10.1016/j.canlet.2016.12.023 28034805

[12] Pol J Kroemer G Galluzzi L First oncolytic virus approved for melanoma immunotherapy. Oncoimmunology (2016) 5(1):e1115641 10.1080/2162402X.2015.1115641 26942095 PMC4760283

[13] Breitbach CJ Lichty BD Bell JC. Oncolytic viruses: therapeutics with an identity crisis. EBioMedicine (2016) 9:31–6. 10.1016/j.ebiom.2016.06.046 27407036 PMC4972563

[14] Msaouel P Opyrchal M Dispenzieri A Peng KW Federspiel MJ Russell SJ Clinical trials with oncolytic measles virus: current status and future prospects. Curr Cancer Drug Targets (2017). 10.2174/1568009617666170222125035 28228086 PMC5630504

[15] Aref S Bailey K Fielding A Measles to the rescue: a review of oncolytic measles virus. Viruses (2016) 8(10):294 10.3390/v8100294 PMC5086626 27782084

[16] Russell SJ Federspiel MJ Peng K-W Tong C Dingli D Morice WG Remission of disseminated cancer after systemic oncolytic virotherapy. Mayo Clin Proc (2014) 89(7):926–33. 10.1016/j.mayocp.2014.04.003 24835528 PMC4225126

[17] Takeuchi K Kadota S-I Takeda M Miyajima N Nagata K Measles virus V protein blocks interferon (IFN)-α/β but not IFN-γ signaling by inhibiting STAT1 and STAT2 phosphorylation. FEBS Lett (2003) 545(2–3):177–82. 10.1016/S0014-5793(03)00528-3 12804771

[18] Devaux P von Messling V Songsungthong W Springfeld C Cattaneo R. Tyrosine 110 in the measles virus phosphoprotein is required to block STAT1 phosphorylation. Virology (2007) 360(1):72–83. 10.1016/j.virol.2006.09.049 17112561

[19] Haralambieva I Iankov I Hasegawa K Harvey M Russell SJ Peng K-W. Engineering oncolytic measles virus to circumvent the intracellular innate immune response. Mol Ther (2007) 15(3):588–97. 10.1038/sj.mt.6300076 17245355 PMC3833616

[20] Campadelli-Fiume G Petrovic B Leoni V Gianni T Avitabile E Casiraghi C Retargeting strategies for oncolytic herpes simplex viruses. Viruses (2016) 8(3):63. 10.3390/v8030063 26927159 PMC4810253

[21] Msaouel P Iankov ID Allen C Russell SJ Galanis E. Oncolytic measles virus retargeting by ligand display. Methods Mol Biol (2012) 797:141–62. 10.1007/978-1-61779-340-0_11 21948475 PMC3691680

[22] Lin L-T Richardson CD. The host cell receptors for measles virus and their interaction with the viral hemagglutinin (H) protein. Viruses (2016) 8(9):E250. 10.3390/v8090250 27657109 PMC5035964

[23] Nakamura T Peng K-W Vongpunsawad S Harvey M Mizuguchi H Hayakawa T Antibody-targeted cell fusion. Nat Biotech (2004) 22(3):331–6. 10.1038/nbt942 14990955

[24] Friedrich K Hanauer JR Prüfer S Münch RC Völker I Filippis C DARPin-targeting of measles virus: unique bispecificity, effective oncolysis, and enhanced safety. Mol Ther (2013) 21(4):849–59. 10.1038/mt.2013.16 23380817 PMC3616535

[25] Nakamura T Peng K-W Harvey M Greiner S Lorimer IA James CD Rescue and propagation of fully retargeted oncolytic measles viruses. Nat Biotech (2005) 23(2):209–14. 10.1038/nbt1060 15685166

[26] Ayala-Breton C Russell LO Russell SJ Peng K-W. Faster replication and higher expression levels of viral glycoproteins give the vesicular stomatitis virus/measles virus hybrid VSV-FH a growth advantage over measles virus. J Virol (2014) 88(15):8332–9. 10.1128/JVI.03823-13 24829351 PMC4135973

[27] Bach P Abel T Hoffmann C Gal Z Braun G Voelker I Specific elimination of CD133+ tumor cells with targeted oncolytic measles virus. Cancer Res (2013) 73(2):865–74. 10.1158/0008-5472.CAN-12-2221 23293278

[28] Radecke F Spielhofer P Schneider H Kaelin K Huber M Dötsch C Rescue of measles viruses from cloned DNA. EMBO J (1995) 14(23):5773–84. 8846771 10.1002/j.1460-2075.1995.tb00266.x PMC394696

[29] Ayala Breton C Wikan N Abbuhl A Smith DR Russell SJ Peng K-W. Oncolytic potency of HER-2 retargeted VSV-FH hybrid viruses: the role of receptor ligand affinity. Mol Ther Oncolytics (2015) 2:15012. 10.1038/mto.2015.12 27119107 PMC4782949

[30] Zimmer G Zimmer K-P Trotz I Herrler G. Vesicular stomatitis virus glycoprotein does not determine the site of virus release in polarized epithelial cells. J Virol (2002) 76(8):4103–7. 10.1128/JVI.76.8.4103-4107.2002 11907250 PMC136080

[31] Manchester M Liszewski MK Atkinson JP Oldstone MB. Multiple isoforms of CD46 (membrane cofactor protein) serve as receptors for measles virus. Proc Natl Acad Sci U S A (1994) 91(6):2161–5. 10.1073/pnas.91.6.2161 8134365 PMC43330

[32] Tatsuo H Ono N Tanaka K Yanagi Y. SLAM (CDw150) is a cellular receptor for measles virus. Nature (2000) 406(6798):893–7. 10.1038/35022579 10972291

[33] Hoffmann M Wu Y-J Gerber M Berger-Rentsch M Heimrich B Schwemmle M Fusion-active glycoprotein G mediates the cytotoxicity of vesicular stomatitis virus M mutants lacking host shut-off activity. J Gen Virol (2010) 91(Pt 11):2782–93. 10.1099/vir.0.023978-0 20631091

[34] Kays S-K Kaufmann KB Abel T Brendel C Bonig H Grez M CD105 is a surface marker for receptor-targeted gene transfer into human long-term repopulating hematopoietic stem cells. Stem Cells Dev (2015) 24(6):714–23. 10.1089/scd.2014.0455 25517513 PMC4356190

[35] Kamentsky L Jones TR Fraser A Bray M-A Logan DJ Madden KL Improved structure, function and compatibility for CellProfiler: modular high-throughput image analysis software. Bioinformatics (2011) 27(8):1179–80. 10.1093/bioinformatics/btr095 21349861 PMC3072555

[36] Lampe J Bossow S Weiland T Smirnow I Lehmann R Neubert W An armed oncolytic measles vaccine virus eliminates human hepatoma cells independently of apoptosis. Gene Ther (2013) 20(11):1033–41. 10.1038/gt.2013.28 23719065

[37] Venugopal C Hallett R Vora P Manoranjan B Mahendram S Qazi MA Pyrvinium targets CD133 in human glioblastoma brain tumor-initiating cells. Clin Cancer Res (2015) 21(23):5324–37. 10.1158/1078-0432.CCR-14-3147 26152745

[38] Waldron NN Kaufman DS Oh S Inde Z Hexum MK Ohlfest JR Targeting tumor-initiating cancer cells with dCD133KDEL shows impressive tumor reductions in a xenotransplant model of human head and neck cancer. Mol Cancer Ther (2011) 10(10):1829–38. 10.1158/1535-7163.MCT-11-0206 21862685 PMC3191276

[39] Prasad S Gaedicke S Machein M Mittler G Braun F Hettich M Effective eradication of glioblastoma stem cells by local application of an AC133/CD133-specific T-cell-engaging antibody and CD8 T cells. Cancer Res (2015) 75(11):2166–76. 10.1158/0008-5472.CAN-14-2415 25840983

[40] Zhu X Prasad S Gaedicke S Hettich M Firat E Niedermann G. Patient-derived glioblastoma stem cells are killed by CD133-specific CAR T cells but induce the T cell aging marker CD57. Oncotarget (2015) 6(1):171–84. 10.18632/oncotarget.2767 25426558 PMC4381586

[41] Hou Y Zou Q Ge R Shen F Wang Y. The critical role of CD133(+)CD44(+/high) tumor cells in hematogenous metastasis of liver cancers. Cell Res (2012) 22(1):259–72. 10.1038/cr.2011.139 21862973 PMC3351911

[42] Hastie E Grdzelishvili VZ. Vesicular stomatitis virus as a flexible platform for oncolytic virotherapy against cancer. J Gen Virol (2012) 93(Pt 12):2529–45. 10.1099/vir.0.046672-0 23052398 PMC4091291

[43] Ozduman K Wollmann G Ahmadi SA van den Pol AN. Peripheral immunization blocks lethal actions of vesicular stomatitis virus within the brain. J Virol (2009) 83(22):11540–9. 10.1128/JVI.02558-08 19726512 PMC2772672

[44] Muik A Stubbert LJ Jahedi RZ Geibeta Y Kimpel J Dold C Re-engineering vesicular stomatitis virus to abrogate neurotoxicity, circumvent humoral immunity, and enhance oncolytic potency. Cancer Res (2014) 74(13):3567–78. 10.1158/0008-5472.CAN-13-3306 24812275

[45] Mrkic B Pavlovic J Rülicke T Volpe P Buchholz CJ Hourcade D Measles virus spread and pathogenesis in genetically modified mice. J Virol (1998) 72(9):7420–7. 9696838 10.1128/jvi.72.9.7420-7427.1998 PMC109970

[46] Myers R Harvey M Kaufmann TJ Greiner SM Krempski JW Raffel C Toxicology study of repeat intracerebral administration of a measles virus derivative producing carcinoembryonic antigen in rhesus macaques in support of a phase I/II clinical trial for patients with recurrent gliomas. Hum Gene Ther (2008) 19(7):690–8. 10.1089/hum.2008.035 18576918 PMC2748764

[47] Ayala-Breton C Suksanpaisan L Mader EK Russell SJ Peng K-W. Amalgamating oncolytic viruses to enhance their safety, consolidate their killing mechanisms, and accelerate their spread. Mol Ther (2013) 21(10):1930–7. 10.1038/mt.2013.164 23842448 PMC3808140

[48] Yanagi Y Takeda M Ohno S. Measles virus: cellular receptors, tropism and pathogenesis. J Gen Virol (2006) 87(10):2767–79. 10.1099/vir.0.82221-0 16963735

[49] Jurgens EM Mathieu C Palermo LM Hardie D Horvat B Moscona A Measles fusion machinery is dysregulated in neuropathogenic variants. MBio (2015) 6(1):e02528–14. 10.1128/mBio.02528-14 25670774 PMC4337580

[50] Watanabe S Shirogane Y Suzuki SO Ikegame S Koga R Yanagi Y. Mutant fusion proteins with enhanced fusion activity promote measles virus spread in human neuronal cells and brains of suckling hamsters. J Virol (2013) 87(5):2648–59. 10.1128/JVI.02632-12 23255801 PMC3571373

[51] Cathomen T Mrkic B Spehner D Drillien R Naef R Pavlovic J A matrix-less measles virus is infectious and elicits extensive cell fusion: consequences for propagation in the brain. EMBO J (1998) 17(14):3899–908. 10.1093/emboj/17.14.3899 9670007 PMC1170725

[52] van den Pol AN Mao G Chattopadhyay A Rose JK Davis JN. Chikungunya, influenza, Nipah, and Semliki forest chimeric viruses with vesicular stomatitis virus: actions in the brain. J Virol (2017) 91(6):e02154–16. 10.1128/JVI.02154-16 28077641 PMC5331823

[53] Wollmann G Drokhlyansky E Davis JN Cepko C van den Pol AN. Lassa-vesicular stomatitis chimeric virus safely destroys brain tumors. J Virol (2015) 89(13):6711–24. 10.1128/JVI.00709-15 25878115 PMC4468483

[54] Hayashi T Ding Q Kuwahata T Maeda K Miyazaki Y Matsubara S Interferon-alpha modulates the chemosensitivity of CD133-expressing pancreatic cancer cells to gemcitabine. Cancer Sci (2012) 103(5):889–96. 10.1111/j.1349-7006.2012.02235.x 22320450 PMC7659312

[55] Yamamuro S Sano E Okamoto Y Ochiai Y Ohta T Ogino A Antitumorigenic effect of interferon-beta by inhibition of undifferentiated glioblastoma cells. Int J Oncol (2015) 47(5):1647–54. 10.3892/ijo.2015.3165 26397698 PMC4599190

[56] Happold C Roth P Silginer M Florea A-M Lamszus K Frei K Interferon-beta induces loss of spherogenicity and overcomes therapy resistance of glioblastoma stem cells. Mol Cancer Ther (2014) 13(4):948–61. 10.1158/1535-7163.MCT-13-0772 24526161

[57] Naniche D Yeh A Eto D Manchester M Friedman RM Oldstone MB. Evasion of host defenses by measles virus: wild-type measles virus infection interferes with induction of Alpha/Beta interferon production. J Virol (2000) 74(16):7478–84. 10.1128/JVI.74.16.7478-7484.2000 10906201 PMC112268

