# Supplementary material for: Enhancing the Oncolytic Activity of CD133-Targeted Measles Virus: Receptor Extension or Chimerism with Vesicular Stomatitis Virus Are Most Effective
Source: Front Oncol. 2017 Jun 26;7:127. doi: 10.3389/fonc.2017.00127 (PMC5483446; doi:10.3389/fonc.2017.00127)
Supplement: Supplementary file 1 [file Data_Sheet_1.DOCX]

Supplementary Material

**Oncolytic viruses retargeted to the tumor stem cell marker CD133**

Dina Kleinlützum^1,2^, Julia D.S. Hanauer^1^, Alexander Muik^1^, Kay-Martin Hanschmann^3^, Sarah-Katharina Kays^1^, Camilo Ayala-Breton^4^, Kah-Whye Peng^4^, Michael D. Mühlebach^5^, Tobias Abel^1^, Christian J. Buchholz^1,2^*

^1^Molecular Biotechnology and Gene Therapy, Paul-Ehrlich-Institut, Langen, Germany;

^2^German Cancer Consortium, Heidelberg; ^3^Biostatistics, Paul-Ehrlich-Institut, Langen, Germany;

^3^Biostatistics, Paul-Ehrlich-Institut, Langen, Germany;

^4^Department of Molecular Medicine, Mayo Clinic, Rochester, Minnesota, USA;

^5^Product Testing of Immunological Medicinal Products for Veterinary Uses, Paul-Ehrlich-Institut, Langen, Germany

*** Correspondence:** Christian J. Buchholz: [christian.buchholz@pei.de](mailto:christian.buchholz@pei.de)

# Supplementary Figures

**Supplementary Figure 1.** Cell surface expression of CD46 and CD133 on human CD34^+^ cells. Stimulated CD34-positive cells isolated from G-CSF mobilized peripheral blood were analyzed for their cell surface expression levels of CD133 (AC133) and CD46 by flow cytometry using FITC- or APC-labeled antibodies. Percent of receptor positive cells for (A) CD46 and (B) CD133 are shown (red lined histograms) in relation to cells stained with Mouse IgG1 isotype control antibodies (gray-filled histograms).

**Supplementary Figure 2.** Dose-response curves of oncolytic viruses on HuH7 cells. Cells were infected with a broad range of MOIs with the indicated viruses. Cell viability was determined 72 hours post infection by the WST-1 cell proliferation assay (Clontech, Germany). Percentage of cell death was calculated against untreated cells followed by subtraction of 100. Based on these response curves EC_50_ values shown in Fig. 3B were determined. Depicted are three independent identically conducted experiments each consisting of four technical replicates.

**Supplementary Figure 3.** Dose-response curves of the indicated oncolytic viruses on NCH644 glioma sphere cultures. Glioma spheres were dissociated into a single-cell suspension and infected with a broad range of MOIs. Cell viability was determined at the indicated time points post infection by RealTime-Glo MT Cell Viability assay (Promega, Germany). Each value is the mean of three technical replicates.

**Supplementary Figure 4.** Dose-response curves of 5-FC mediated cell killing of MV^SCD^-CD133 on NCH644 glioma spheres. Glioma sphere cells were dissociated to a single-cell suspension and infected with MV-CD133 or MV^SCD^-CD133 at an MOI of 1. Cells were cultivated in presence of different concentrations of 5-FC and their viability was quantified by RealTime-Glo MT Cell Viability assay (Promega, Germany). Depicted are three technical replicates of dose-response curves obtained at 72 hours post infection.
